# Fatal Case of Mediterranean Spotted Fever Associated with Septic Shock, Iran

**DOI:** 10.3201/eid2802.211023

**Published:** 2022-02

**Authors:** Saber Esmaeili, Mina Latifian, Mohammad Khalili, Mehrdad Farrokhnia, John Stenos, Mehdi Shafiei, Ehsan Mostafavi

**Affiliations:** Pasteur Institute of Iran, Tehran, Iran (S. Esmaeili, M. Latifian, E. Mostafavi);; Pasteur Institute of Iran, Akanlu, Iran (S. Esmaeili, M. Latifian, E. Mostafavi);; Shahid Bahonar University of Kerman, Kerman, Iran (M. Khalili);; Kerman University of Medical Sciences, Kerman (M. Farrokhni, M. Shafiei);; University Hospital Geelong, Geelong, Victoria, Australia (J. Stenos)

**Keywords:** Mediterranean spotted fever, Iran, zoonoses, vector-borne infections, Rickettsia, *Rickettsia conorii*, bacteria

## Abstract

A fatal case of Mediterranean spotted fever associated with septic shock was reported in a 61-year-old man living in a village in southeastern Iran. The patient had a history of tick bite a few days before symptom onset. Phylogenetic analysis confirmed infection by *Rickettsia conorii* subspecies *israelensis*.

Mediterranean spotted fever (MSF) is a zoonotic disease caused by *Rickettsia conorii*. The main vector of this bacterium is the *Rhipicephalus sanguineus* tick (*1*); the main hosts of these ticks are domestic dogs, and humans are incidental hosts (*2*). MSF is endemic to the Mediterranean, Europe, Africa, western Asia, and India. The case-fatality rate is 3%–7% in hospitalized patients (*3*,*4*).

In 2017, human cases of MSF were reported in Kerman province in southeastern Iran (*5*). No data are available on the epidemiology of MSF in Iran; we report a fatal case of MSF associated with septic shock.

The patient was a 61-year-old man with a 10-year history of hypertension and rheumatoid arthritis who lived in a village in proximity to Bam County, Kerman province, Iran. He was a farmer, had no history of domestic animal-keeping, and reported contact with livestock and a tick bite a few days before symptom onset. The initial clinical signs of the disease appeared on September 6, 2019, and the patient was admitted to a hospital in Bam on September 9; symptoms were fever, nausea, vomiting, myalgia, urinary retention, and flank pain. The patient had scleral icterus, and a black skin eschar at the tick bite site and skin rash were visible on his left leg.

When the patient’s condition deteriorated, he was transferred to a hospital in Kerman on September 15. At admission, symptoms were septic shock, tachycardia, tachypnea, fever, and hypotension (85/50 mm Hg); he immediately began treatment with ceftriaxone, metronidazole, and parenteral hydration. Maculopapular skin rash was visible on the left leg. The patient had thrombocytopenia, and an increase was observed in leukocyte counts, renal factor levels (urea and creatinine), liver enzyme levels (aspartate aminotransferase, alanine transferase, and alkaline phosphatase), partial thromboplastin time of coagulation, and bilirubin levels (Table). Hemoglobin and hematocrit levels decreased, and the patient experienced hematuria and proteinuria; calcium oxalate and amorphous urate crystals were further reported in microscopic examinations. Treatment of prednisolone, heparin, doxycycline, and vancomycin was initiated.

**Table:** Laboratory findings in a patient with Mediterranean spotted fever associated with septic shock, Iran*

Value	2019 Sep 16, 12 AM	2019 Sep 17, 1 AM	2019 Sep 17, 1 AM
Leukocyte, × 10^9^/L	18,900	12,700	ND
Hemoglobin, g/dL	12.9	14.1	ND
Platelets, × 10^9^/L	56,000	42,000	ND
Hematocrit, %	35.3	43.0	ND
Prothrombin time, s	14.4	13.5	14
Partial thromboplastin time, s	56	39	33
Aspartate aminotransferase, U/L	83	101	ND
Alanine aminotransferase, U/L	71	49	ND
Alkaline phosphatase, U/L	328	510	ND
Bilirubin total, mg/dL	2.7	4.8	ND
Bilirubin direct, mg/dL	2.3	2.8	ND
Blood urea, mg/dL	95	145	161
Blood creatinine, mg/dL	3.6	4.8	5.5
Blood calcium, mEq/L	8.5	ND	ND
Blood sodium, mEq/L	140	135	136
Blood potassium, mEq/L	4.0	4.9	3.5
Proteinuria	+	–	+
Hematuria	+	–	+

*ND, not done; +, positive; –, negative.

On September 16, the patient was transferred to Afzalipour Hospital in Kerman (Referral Center for Infectious Diseases, Kerman Province). At the time of admission, the patient was conscious, his condition was stable, and his temperature was 37.6°C. No abnormalities were observed in clinical examinations of the heart, chest, and abdomen, but we noted bilateral lower extremity edema and left leg skin lesions (rash and eschar). The results of laboratory tests of blood and urine samples were abnormal (Table). The patient underwent emergency dialysis and continued to take prednisolone, heparin, doxycycline, and vancomycin. On September 17, the patient lost consciousness; he was subsequently intubated and admitted to the intensive care unit. A few hours later, he experienced septic shock and cardiac arrest and died.

The differential diagnosis for this patient included MSF and Crimean-Congo hemorrhagic fever; on September 17, samples required for these differential diagnoses were prepared. Serum and blood samples were sent to the Pasteur Institute of Iran on September 25 (8 days after the patient’s death). Serologic and molecular test results for Crimean-Congo hemorrhagic fever were negative. Testing for *R. conorii* IgM by ELISA was borderline, and titer of *R. conorii* IgM by immunofluorescence assay was 1:48. Serum samples were positive for *Rickettsia* spp. (16S rRNA gene) by real-time reverse transcription PCR (*6*). On the basis of the amplification and sequencing of specific genes of *Rickettsia* spp. (*gltA*, GenBank accession no. MZ545594.1; *17KD*, GenBank accession no. MZ545592.1; *ompA*, GenBank accession no. MZ545593.1), we confirmed infection by *R. conorii* subspecies *israelensis* (Figure).

**Figure:**
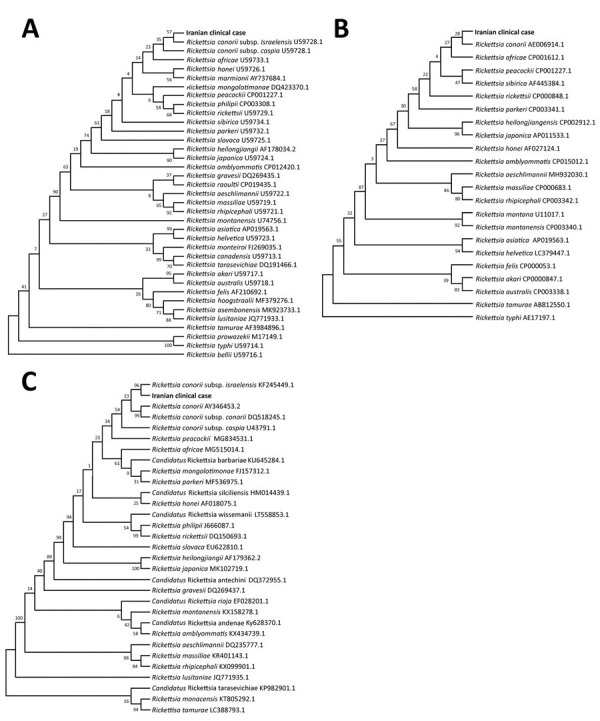
Phylogenetic analysis of *Rickettsia* species from a patient with Mediterranean spotted fever associated with septic shock, Iran (in bold), confirming infection with *R. conorii* subspecies *israelensis*. A) gltA gene; B) *17KD* gene; C) *ompA* gene. Tree was constructed with the maximum-likelihood method algorithm (Tamura-Nei model). The test was performed with bootstrap (500 repetitions) by MEGA X 10.1 software (https://www.megasoftware.com).

The patient died as a result of late diagnosis of a rickettsial infection and subsequent septic shock, despite initiation of appropriate treatment. MSF is usually considered to be a mild disease, but severe and fatal cases do occasionally occur (*7*). One of the causes of death from MSF is multiorgan failure, including acute kidney injury, pneumonitis, and encephalitis. When severe, MSF can manifest as septic shock, and acute kidney injury might occur. Thrombocytopenia and elevated liver enzymes are frequent laboratory abnormalities (*4*,*7*).

Phylogenetic trees showed that the infection in this patient was caused by *R. conorii* subsp. *israelensis*. *R. conorii* has 4 subspecies, *caspia*, *israelensis*, *conorii*, and *indica*, each of which cause diseases that have specific clinical features and occur in different geographic regions. *R. conorii* subsp. *israelensis* seems to have the highest death rate of the subspecies (*8*,*9*), reported to be ≈30% (*10*).

MSF appears to be circulating in southern Iran but is a neglected disease that requires more attention from the healthcare system. Because of the nonspecific clinical symptoms of MSF, diagnosing the disease is challenging. Diagnosing and treating MSF early is critical to prevent progression to more severe illness (*6*). Further studies, particularly on elucidating potential reservoirs and vectors, will result in a better understanding of the epidemiology of this disease in Iran. In the meantime, MSF should be included in the differential diagnosis for patients in Iran who are experiencing fever and rash.
